# Overweight and Obesity in Finnish Children by Parents’ Socioeconomic Position—A Registry-Based Study

**DOI:** 10.3389/ijph.2023.1605901

**Published:** 2023-09-01

**Authors:** Päivi Mäki, Esko Levälahti, Susanna Lehtinen-Jacks, Tiina Laatikainen

**Affiliations:** ^1^ Health and Well-Being Promotion Unit, Finnish Institute for Health and Welfare (THL), Helsinki, Finland; ^2^ The Health Sciences Unit, Faculty of Social Sciences, Tampere University, Tampere, Finland; ^3^ Division of Public Health Sciences, School of Health, Care and Social Welfare, Mälardalen University, Västerås, Sweden; ^4^ Institute of Public Health and Clinical Nutrition, University of Eastern Finland, Kuopio, Finland

**Keywords:** childhood obesity, socioeconomic position, registry-based monitoring, health disparities, overweight and obesity

## Abstract

**Objectives:** To examine associations between parents’ socioeconomic position (SEP) and child overweight and obesity, using registry data.

**Methods:** Data (final *n* = 194,423) on children’s height, weight and parents’ SEP were drawn from the national Register of Primary Health Care Visits (Avohilmo) and Statistics Finland. Risk ratios for bernoulli-distributed overweight (RR_OW_) and obesity (RR_OB_) according to SEP were estimated using generalized linear models and using a log -link.

**Results:** The risk for obesity was lower in boys from high-income families (RR_OB_ 0.76), for overweight and obesity was lower in boys (RR_OW_ 0.72, RR_OB_ 0.58) and girls (RR_OW_ 0.72, RR_OB_ 0.54) with highly educated fathers, in boys (RR_OW_ 0.79, RR_OB_ 0.58) and girls (RR_OW_ 0.78, RR_OB_ 0.56) with high-educated mothers and in boys (RR_OW_ 0.85, RR_OB_ 0.77) and girls (RR_OW_ 0.80, RR_OB_ 0.69) living in urban areas, as compared to low-income families, low-educated parents, and rural residence, respectively.

**Conclusion:** The risk of overweight and obesity was increased in children with low SEP or rural residence. Administrative registers are a valid approach to monitor childhood obesity by parents’ SEP.

## Introduction

Childhood obesity is a major public health problem globally [1]. In Finland, recent statistics showed that 29% of boys aged 2–16 years and 18% of girls were living with overweight (including obesity), and 9% of boys and 4% of girls with obesity [2].

Childhood obesity tends to continue into adulthood [3]. Excess weight is associated with several physical and psychosocial health concerns in childhood and later in adulthood [4]. The majority of children with obesity also have other risk factors for arterial diseases, and the risk increases as obesity becomes more severe [4–7]. In addition, children living with obesity are more likely to suffer bullying, social exclusion, low self-esteem, and body image dissatisfaction than their peers with healthy weight [8, 9].

There are several individual and societal factors behind obesity in children, such as heredity, lifestyle habits and obesogenic living environment [4, 10]. Previous studies have shown that obesity is associated with socioeconomic position (SEP), both in adults and children [11, 12]. Parents’ education has a strong, inverse association with childhood obesity in high-income countries, but the opposite relationship in most of the middle-income and low-income countries [12, 13]. In general, children with low SEP in high-income countries and children with high SEP in low-income countries are at higher risk of overweight than other SEP groups [12, 14].

Finnish administrative registers include information on measured height and weight of children and parents’ socioeconomic position. In a previous study the association of a large set of registry-based indicators on parents’ SEP and childhood obesity was analyzed and parents’ education and the household’s disposable monetary income were found to be the variables most strongly associated with obesity in children [15].

In the present study we had two aims: 1) to examine associations between parents’ SEP variables, which were most strongly associated with obesity in their offspring [15], and the prevalence of overweight and obesity in children aged 2–17 years, based on data from two Finnish administrative registers; and 2) to discover if linked, registry-based data on children’s height and weight and parents’ SEP could be used to monitor national childhood obesity.

## Methods

### Study Population

Data on children’s height and weight were extracted from the Register of Primary Health Care Visits (Avohilmo), maintained by the Finnish Institute for Health and Welfare (THL) [16]. Avohilmo includes real-time data on primary healthcare visits, including measured height and weight, collected via automatic data transmission from electronic health records of primary health care units. The data extraction criteria were that a child had visited a child health clinic, school health care or student health care between 1st of January 2018 and 31st of December 2018 and both height and weight were recorded during the visit (*n* = 397,047). Indicators extracted for the current study included date of birth, sex, age and height and weight measurements with their respective measurement dates. The exact age at measurement was calculated based on the child’s date of birth and the date of health care visit.

The data were validated as follows; 1) deviation statistics for height and weight values were calculated using the Finnish growth standard as reference values (relative to weight by sex and height, height by sex and age, body mass index (BMI, kg/m^2^) by sex and age, children’s BMI corresponding to the adult BMI (ISO-BMI kg/m^2^) [17], 2) children with deviation values outside the [-4,4] boundary were excluded, 3) height and weight measurements resulting in ISO-BMI ≥50 were excluded. In addition, 1,537 children were excluded because valid height and weight measurements were not available in 2018. As a result, the data included 395,510 children who were 2–17 years old and had at least one valid height and weight measurement in 2018.

Avohilmo’s data on children’s height and weight were linked on an individual level with Statistics Finland’s data from 1 January 2014 to 31 December 2018 on adults’ (both parents’) socioeconomic position (SEP) living in the same address as a child using deterministic linkage and personal identification code.

Data on parents’ SEP were not found for 7,887 children. In addition, siblings and half-siblings and families with two female or two male adults were excluded from the analysis (*n* = 193,200). As a result, each parent was included in the data only once, as was only one child from each family. The number of families with two female or two male adults was 374. The final data included 194,423 (100,216 boys and 94,207 girls) children with measured height and weight and information on parents’ SEP.

### Definition of Overweight and Obesity

Overweight and obesity were defined according to the WHO growth reference for children [18, 19]. For children from two to five years of age, the definition for overweight and obesity were a BMI-for-age value greater than +2 SD and +3 SD above the WHO Child Growth Standard median, respectively [18, 20]. For children over 5 years old, overweight and obesity were defined as a BMI-for-age value greater than +1 SD and +2 SD above the WHO Growth Reference median, respectively [19, 21].

The prevalence of overweight (including obesity) and obesity in boys and girls by parents’ SEP are presented in Supplementary Tables S1, S2.

### Indicators of Socioeconomic Position

The following parents’ SEP indicators were selected for analyses; mother’s and father’s educational level of highest qualification/degree, household’s disposable monetary income, and in addition, the father’s and mother’s age and municipality group of municipality of domicile according to the 2016 regional division.

The SEP variables had several categories. For the analysis the SEP variables were re-categorized as follows: 1) Parental age was classified into six categories: <30, 30–34, 35–39, 40–44, 45–49, ≥50 years and 2) child’s age classified into four categories: 2–6.99, 7–12.99, 13–15.99 and 16–17.99 years. 3) Father’s and mother’s educational level of highest qualification/degree was classified into three categories: low, medium and high. The category of low education included parents who did not have a degree, i.e., they had completed at most primary school. The category of medium level of education included parents who had completed secondary or high school or vocational school. The category of high education included parents who had a bachelor’s degree, master’s degree or higher degree. 4) Household’s disposable monetary income was calculated considering the size of the household as follows: Each household’s yearly disposable monetary income/12/weighting coefficient. Weighting coefficient: 1.0 to the first adult, 0.5 to the second and each subsequent person aged 14 years and over, 0.3 to each child aged under 14 years, 0.4 to each child of unknown age [22]. Household’s disposable monetary income was classified into three categories: low (<1,525 euros per month), middle (from 1,525 to 4,065 euros per month) and high (>4,065 euros per month). 5) Municipality of domicile according to the 2016 regional division was classified into three categories: urban, semi-urban and rural municipalities. In urban municipalities at least 90% of the population lives in urban settlements or the population of the largest urban settlement is at least 15,000. In semi-urban municipalities, 60%–90% of the population lives in urban settlements and the population of the largest urban settlement is at least 4,000 but less than 15,000. In rural municipalities, less than 60% of the population lives in urban settlements and the population of the largest urban settlement is less than 15,000 or 60%–90% of the population lives in urban settlements and in which the population of the largest settlement is less than 4,000 [23]. Data characteristics are shown in the Table 1.

**TABLE 1:** Data characteristics, %. (A registry-based study, Finland, 2016–2018).

	Boys				Girls			
Age group (years)	2–6.99	7–12.99	13–15.99	16–17.99	2–6.99	7–12.99	13–15.99	16–17.99
N	34,814	36,229	17,270	11,903	33,459	34,619	16,720	9,409
Overweight^a^ ^,^ ^b^	14.2	35.4	30.8	29.0	13.3	28.7	27.6	25.4
Obesity^a^	4.2	14.8	12.6	11.6	3.4	8.9	8.2	7.7
Father’s age (years)								
<30	12.2	2.3	0.5	0.1	12.4	2.4	0.5	0.2
30–34	27.9	10.4	2.1	0.8	27.3	10.3	2.4	1.0
35–39	32.0	24.3	9.0	4.0	32.4	24.3	9.6	4.4
40–44	17.8	30.2	23.5	14.4	18.0	30.1	23.4	15.1
45–49	6.7	19.2	30.2	29.4	6.5	19.2	29.5	30.1
≥50	3.4	13.7	34.5	51.4	3.3	13.6	34.7	49.2
Missing (n)	3,073	5,174	3,017	2,220	2,938	5,051	3,005	1,789
Mother’s age (years)								
<30	22.4	4.8	0.2	0.1	22.1	4.5	0.2	0.1
30–34	32.2	16.3	4.2	0.8	32.7	16.3	4.3	1.1
35–39	29.6	29.1	14.1	7.4	29.3	29.1	15.0	7.8
40–44	13.0	29.2	29.3	21.0	13.0	29.4	29.2	22.5
45–49	2.6	15.7	30.7	33.5	2.7	15.5	30.6	33.7
≥50	0.2	4.9	21.5	37.2	0.2	5.1	20.8	34.9
Missing (n)	113	800	586	468	98	582	517	301
Household’s disposable monetary income								
Low	28.5	23.7	19.4	16.0	27.8	23.9	19.7	16.6
Middle	67.7	70.8	74.0	76.0	68.2	70.4	73.5	75.6
High	3.7	5.5	6.6	8.0	4.0	5.7	6.8	7.8
Missing (n)	319	1,032	714	593	293	782	514	355
Father’s degree of highest education								
Low	13.2	13.3	14.0	12.9	13.0	13.2	13.7	14.2
Middle	45.4	45.1	44.8	45.6	46.1	44.7	45.4	43.4
High	41.3	41.6	41.1	41.5	40.9	42.1	40.8	42.3
Missing (n)	2,693	4,978	2,951	2,178	2,562	4,896	2,930	1,747
Mother’s degree of highest education								
Low	10.4	9.2	8.9	8.0	10.2	8.9	8.8	9.2
Middle	37.1	36.8	37.4	37.5	36.7	36.7	37.9	37.6
High	52.4	54.0	53.7	54.5	53.1	54.4	53.3	53.2
Missing (n)	112	798	584	468	95	582	516	301
Municipality group								
Urban	75.9	73.7	71.7	70.9	75.4	73.8	71.6	71.1
Semi-urban	14.6	15.4	16.9	16.5	14.7	15.5	16.8	16.2
Rural	9.5	10.9	11.4	12.6	9.9	10.7	11.6	12.6

^a^ Overweight and obesity were defined according to the WHO growth reference for children [18, 19].

^b^ The prevalence of overweight includes obesity.

### Statistical Analyses

Before categorizing, most of the SEP indicators were imputed. If no previous individual data were available (preferably in 2017 or secondly in 2014–2016), data from 2018 were used. Parents’ education was imputed using values from previous years. For continuous predictors, individual trends were fitted and used for prediction of missing values. For continuous predictor data with only one observation, one trend line was fitted and used for prediction of missing values.

The analyses were carried out using a randomly selected training data (*n* = 155,479, 80% of the data; 80,216 boys and 75,263 girls) and left out data (*n* = 38,944, 20% of the data; 20,000 boys and 18,944 girls), which was used for model testing and validation.

Risk ratios for bernoulli-distributed overweight (RR_OW_) and obesity (RR_OB_) according to SEP were estimated using generalized linear models and using the log -link function.

### Model Fitting

Generalized linear models for overweight (including obesity) and obesity were fitted for boys and girls separately. The effects of four predictor indicators (father’s education, mother’s education, household’s disposable monetary income and municipality group of municipality of domicile) were analyzed by adjusting for child’s, mother’s and father’s age. As a first step, the univariate effect of four predictor indicators were analyzed using adjustments. In the second step, alternative multivariate main effect models were analyzed. Finally, all predictor and adjusting indicators were tested for interactions.

### Multivariate Model Fitting and Testing

The following alternative multivariate models were fitted: 1. Full model of main effects (four predictors and three adjusting indicators), 2. Models to exclude one of the predictors (four submodels), 3. Models to include one twoway interaction between predictors or between one predictor and one adjusting indicator (18 submodels).

All models were fitted in training data and submodels were tested in left out data. Deviance residuals were predicted for full model and all submodels. The sum of squared deviance residuals (D) in left out data was used as a test statistic to test the model against full model. The difference of D-statistics were considered as chi-square distributed with the number of freely estimated parameters dropped as degrees of freedom.

Chi-square statistic for main effects: Chi-square = (D-statistic of the full model of main effects) - (D-statistic of submodel).

Chi-square statistic for interactions: Chi-square = (D-statistic of model with one interaction) - (D-statistic of the full model of main effects).

The final model was validated by comparing Area under the ROC curve (AUC) in training and left out data.

The nonsignificant result from the test for equality of AUC between training and left out data was considered a valid model for obesity or overweight.

Stata/MP 17.0 statistical software was used for all data management and statistical analyses. The significance level was 0.001 for all tests.

## Results

The prevalence of overweight and obesity was higher in boys than in girls and in older children compared to younger children. In total, 31% of boys and 24% of girls had overweight or obesity. Overweight (including obesity) and obesity were the most common in 7–13-year-old boys and girls (in boys 35% and 15%, in girls 29% and 9%, respectively) (Supplementary Tables S1, S2).

### Association of Overweight and Obesity and Parents’ SEP Indicators

For boys and girls, all main effects were significant for obesity and overweight models, except for household’s disposable monetary income for girls’ overweight and obesity models. None of the tested interactions were significant. All final models were valid with no significant differences in AUCs between training and left out data (Tables 2–5).

**TABLE 2:** Generalized linear models for overweight in boys. (A registry-based study, Finland, 2016–2018).

	Univariate RR (99.9% CI)	Multivariate RR (99.9% CI)
Household’s disposable monetary income		
Low	1 (ref)	1 (ref)
Middle	0.98 (0.94–1.03)	1.08 (1.03–1.14)
High	0.76 (0.68–0.84)	0.95 (0.86–1.06)
Missing	1.15 (1.03–1.29)	1.26 (1.11–1.43)
Father’s degree of highest education		
Low	1 (ref)	1 (ref)
Middle	0.91 (0.86–0.96)	0.92 (0.86–0.97)
High	0.66 (0.62–0.70)	0.72 (0.68–0.77)
Missing	0.94 (0.74–1.20)	0.96 (0.76–1.23)
Mother’s degree of highest education		
Low	1 (ref)	1 (ref)
Middle	0.96 (0.90–1.02)	0.95 (0.89–1.01)
High	0.73 (0.69–0.78)	0.79 (0.74–0.84)
Missing	0.98 (0.84–1.15)	0.88 (0.74–1.06)
Municipality group		
Rural	1 (ref)	1 (ref)
Semi-urban	0.94 (0.88–1.00)	0.96 (0.90–1.03)
Urban	0.79 (0.75–0.84)	0.85 (0.80–0.89)

AUC for left out data: 0.655, AUC for training data: 0.662, model validation test *p*-value = 0.124 Adjustment for child’s, mother’s and father’s age. CI, confidence interval; RR: risk ratio.

**TABLE 3:** Generalized linear models for overweight in girls. (A registry-based study, Finland, 2016–2018).

	Univariate RR (99.9% CI)	Multivariate RR (99.9% CI)	Multivariate (best fitting model) RR (99.9% CI)
Household’s disposable monetary income			
Low	1 (ref)	1 (ref)	
Middle	0.94 (0.89–0.99)	1.05 (0.99–1.11)	
High	0.72 (0.64–0.81)	0.94 (0.83–1.06)	
Missing	1.14 (0.98–1.31)	1.33 (1.14–1.55)	
Father’s educational level of highest qualification/degree			
Low	1 (ref)	1 (ref)	1 (ref)
Middle	0.92 (0.86–0.98)	0.94 (0.87–1.00)	0.94 (0.88–1.01)
High	0.63 (0.59–0.68)	0.72 (0.66–0.77)	0.72 (0.66–0.77)
Missing	1.05 (0.78–1.41)	1.10 (0.82–1.49)	1.10 (0.82–1.49)
Mother’s educational level of highest qualification/degree			
Low	1 (ref)	1 (ref)	1 (ref)
Middle	0.95 (0.89–1.02)	0.95 (0.88–1.02)	0.95 (0.89–1.02)
High	0.70 (0.65–0.75)	0.77 (0.71–0.83)	0.78 (0.72–0.84)
Missing	0.87 (0.71–1.06)	0.76 (0.61–0.95)	0.90 (0.73–1.10)
Municipality group			
Rural	1 (ref)	1 (ref)	1 (ref)
Semi-urban	0.89 (0.82–0.96)	0.92 (0.85–0.99)	0.92 (0.85–0.99)
Urban	0.75 (0.70–0.80)	0.80 (0.75–0.85)	0.80 (0.75–0.85)

AUC for left out data: 0.634, AUC for training data: 0.644, model validation test *p*-value = 0.045 Adjustment for child’s, mother’s and father’s age. CI, confidence interval; RR, risk ratio.

**TABLE 4:** Generalized linear models for obesity in boys. (A registry-based study, Finland, 2016–2018).

	Univariate RR (99.9% CI)	Multivariate RR (99.9% CI)
Household’s disposable monetary income		
Low	1 (ref)	1 (ref)
Middle	0.88 (0.81–0.95)	1.07 (0.98–1.16)
High	0.48 (0.39–0.60)	0.76 (0.61–0.94)
Missing	1.14 (0.93–1.39)	1.36 (1.09–1.69)
Father’s educational level of highest qualification/degree^1^		
Low	1 (ref)	1 (ref)
Middle	0.84 (0.76–0.92)	0.88 (0.79–0.97)
High	0.45 (0.41–0.51)	0.58 (0.51–0.65)
Missing	0.93 (0.61–1.42)	0.99 (0.65–1.51)
Mother’s educational level of highest qualification/degree		
Low	1 (ref)	1 (ref)
Middle	0.86 (0.78–0.96)	0.86 (0.77–0.95)
High	0.50 (0.44–0.55)	0.58 (0.51–0.65)
Missing	0.87 (0.66–1.14)	0.76 (0.56–1.04)
Municipality group		
Rural	1 (ref)	1 (ref)
Semi-urban	0.94 (0.83–1.05)	0.98 (0.88–1.10)
Urban	0.69 (0.62–0.76)	0.77 (0.70–0.85)

AUC for left out data: 0.692, AUC for training data: 0.694, model validation test *p*-value = 0.812 Adjustment for child’s, mother’s and father’s age. CI, confidence interval; RR, risk ratio.

**TABLE 5:** Generalized linear models for obesity in girls. (A registry-based study, Finland, 2016–2018).

	Univariate RR (99.9% CI)	Multivariate RR (99.9% CI)	Multivariate (best fitting model) RR (99.9% CI)
Household’s disposable monetary income			
Low	1 (ref)	1 (ref)	
Middle	0.81 (0.73–0.90)	1.00 (0.90–1.12)	
High	0.43 (0.32–0.57)	0.71 (0.54–0.95)	
Missing	1.12 (0.84–1.49)	1.35 (0.98–1.86)	
Father’s educational level of highest qualification/degree			
Low	1 (ref)	1 (ref)	1 (ref)
Middle	0.82 (0.72–0.94)	0.88 (0.77–1.00)	0.88 (0.77–1.00)
High	0.41 (0.35–0.48)	0.55 (0.46–0.64)	0.54 (0.46–0.63)
Missing	1.13 (0.61–2.09)	1.25 (0.67–2.31)	1.24 (0.67–2.30)
Mother’s educational level of highest qualification/degree			
Low	1 (ref)	1 (ref)	1 (ref)
Middle	0.88 (0.76–1.00)	0.89 (0.77–1.02)	0.88 (0.77–1.01)
High	0.46 (0.40–0.53)	0.56 (0.48–0.66)	0.56 (0.48–0.65)
Missing	0.91 (0.61–1.36)	0.82 (0.52–1.29)	0.97 (0.65–1.45)
Municipality group			
Rural	1 (ref)	1 (ref)	1 (ref)
Semi-urban	0.83 (0.72–0.97)	0.88 (0.76–1.02)	0.88 (0.76–1.02)
Urban	0.61 (0.54–0.69)	0.69 (0.61–0.78)	0.69 (0.61–0.78)

AUC for left out data: 0.661, AUC for training data: 0.676, model validation test *p*-value = 0.078 Adjustment for child’s, mother’s and father’s age. CI, confidence interval; RR, risk ratio.

In multivariate models the risk for obesity was lower in boys in families with higher household’s disposable monetary income (RR_OB_ 0.76, 99.9% CI 0.61–0.94) compared to boys in families with low income. The risk for overweight was higher in boys in middle-income families (RR_OW_ 1.08, 99.9% CI 1.03–1.14) compared to boys in low-income families. The risk for overweight and obesity was lower in boys and girls whose father had high education (boys: RR_OW_ 0.72, 99.9% CI 0.68–0.77, RR_OB_ 0.58, 99,9% CI 0.51–0.65; girls: RR_OW_ 0.72 99.9% CI 0.66–0.77, RR_OB_ 0.54 99.9% CI 0.46–0.63, respectively) compared to children with a lowly educated father. Comparison of the highest with the lowest level of mother’s education showed decreased risk for overweight and obesity in boys (boys: RR_OW_ 0.79, 99.9% CI 0.74–0.84, RR_OB_ 0.58, 99.9% CI 0.51–0.65, respectively) and in girls (RR_OW_ 0.78 99.9% CI 0.72–0.84, RR_OB_ 0.56 99.9% CI 0.48–0.65) in children with highly educated mothers (Tables 2–5).

Risk for overweight and obesity was lower in boys and girls living in urban areas (boys: RR_OW_ 0.85, 99.9% CI 0.80–0.89, RR_OB_ 0.77, 99.9% CI 0.70–0.85; girls: RR_OW_ 0.80 99.9% CI 0.75–0.85, RR_OB_ 0.69 99.9% CI 0.61–0.78, respectively) compared with boys and girls in rural areas (Tables 2–5).

## Discussion

Our study, with data from two Finnish administrative registers, showed that overweight and obesity in children were inversely associated with parents’ SEP (household’s disposable monetary income, father’s and mother’s education), i.e., the prevalence of obesity was higher in children with parents with lower SEP. Furthermore, overweight and obesity were more prevalent in children living in rural areas than children living in urban areas. All the observed associations were stronger regarding obesity, as compared to overweight.

Many previous surveys, conducted in high-income countries, have found that the parents’ low SEP is associated with higher risk of childhood obesity [13, 14, 24]. Our registry-based study supports these earlier findings. A similar association between SEP and the prevalence of overweight and obesity in children has previously been observed in Finnish boys and girls, whether using indicators of family SEP [25–27], indicators of adolescent’s own social position (school achievement, school attendance) or indicators of family’s material affluence [26]. In addition, a large, Finnish prospective birth cohort study found that exposure to neighborhood socioeconomic disadvantage (average annual income, unemployment, and level of education) constitutes an important risk factor for the development of childhood obesity [28].

Magnusson et al. [29] concluded in their review that there are social inequalities in overweight and obesity in the Nordic countries, in spite of socially egalitarian ideals and a reputation for low levels of inequality. They discussed that causes behind the social gradient may not be the ones that have made the prevalence rise in the whole population. Furthermore, some factors which affect the whole population may affect population groups with low SEP even more seriously [29]. Reasons for this could be several: people with more resources may have greater potential to avoid the adverse effects of an obesogenic living environment, education increases consciousness on health and healthy lifestyle, higher income increases freedom of choice concerning food and leisure activities [30]. One potential explanatory factor behind the social gradient is stress. Parents’ low SEP may negatively affect the psychosocial security experienced in families, for example due to job insecurity or living in a poorer residential area [31]. Häkkänen et al. found that living in a divorced or single-parent family was related to the development of obesity and living in non-native families was related to the persistence of obesity among girls [32].

In childhood, family and living environment play important roles in adopting, establishing and promoting healthy lifestyles through role modelling and support for engaging in healthy lifestyle habits [33–37]. Previous Finnish studies have shown a socioeconomic gradient between parents’ SEP and lifestyle habits in childhood and adolescence [36, 38–40]. A systematic review identified several factors, such as parental obesity, child-care attendance, and high TV viewing time, which mediate the relationship between SEP and childhood overweight and obesity [41]. The authors of the review concluded that families from different SEP groups had different risk and protective factors for children’s obesity. Regarding families with low SEP, parental obesity and maternal depressive symptoms were strong risk factors for overweight and obesity in children, whereas long maternal working hours and a permissive parenting style were risk factors for families with higher SEP [41]. In addition, social disadvantage affects families for several generations [42]. Children in families with low SEP are more likely to become adults with low SEP who accumulate less wealth to pass on to future generations [43].

In line with previous research, we observed that overweight and obesity were more common in children living in rural areas than in urban areas [25, 29, 44, 45]. Similar results have been found in Sweden, where overweight and obesity in 6 to 9 year-old children were approximately 2 and 3 times more prevalent, respectively, in areas with lower education than in areas with higher level of education. These associations were explained by lower educational attainment in the rural areas [29]. In our study, the risk for children’s overweight and obesity in urban vs. rural areas diluted when adjusted for parents’ education and household income but did not disappear completely. Hence, other explanations are needed. An explanation could be that children have fewer opportunities for physical activity (long distances to school and forums for sports and other organized physical activity, fewer opportunities to use public transport, fewer alternatives for active transport on foot or by bicycle, safety concerns) in rural areas [46].

In our study, the associations between parent’s SEP and overweight and obesity in children were quite similar in boys and girls in different age groups. Although the results, by indicators we used, were quite consistent, the association may still be more complex than that. According to a systematic review, health behaviors contribute to the association between SEP and health outcomes, but the contribution varies according to geographic location, sex, age, health outcomes and methodological differences between studies [47]. The association between SEP and obesity also varies by several demographic (e.g., age, sex, ethnicity) or environmental (e.g., countries, urban/rural) factors [12]. Furthermore, each SEP indicator has a different background and measures different, often interrelated aspects of socioeconomic stratification. Different SEP indicators may also be more or less relevant to different health outcomes and at different stages of life [13, 48]. Finally, children do not have a degree of education, occupation, or income of their own [13], and regarding adolescents, Koivusilta et al. [26] have stressed that health differences between adolescents are an outcome of several mechanisms, not a direct result of socioeconomic inequality between families. They mentioned that adolescents are experiencing a transition from being children living under parents’ care to being more like independent actors in a wider society. At that stage of life, adolescents’ schooling, education, and family’s material commodities are important because they reflect the adolescents’ own social position and standard of living. Health differences may begin to increase when problems accumulate and intertwine in these spheres of life, for example, because of economic problems or inadequate social networks [26].

Finland has a comprehensive public health care system and almost all families with children use the child health clinic and school health care services, irrespective of their socioeconomic background [49, 50]. This provides an excellent possibility to discuss lifestyle habits, to monitor children’s growth and to identify children with a high risk of obesity. In addition, regular growth monitoring of all children enables national monitoring of overweight and obesity in children, because the data on height and weight are transferred to the national administrative register Avohilmo. Avohilmo has previously been found to be a reliable source for monitoring the prevalence of overweight and obesity in children [51, 52]. Accordingly, the prevalence of overweight and obesity in children has been reported annually at the national, regional and municipal level as part of the reporting of the FinChild register since 2018 [2].

In this study, we linked data from the Avohilmo register to another administrative register, Statistic Finland, to investigate associations between parents’ SEP and overweight and obesity in children. The possibility for individual-level linkage of register data and availability of various indicators of socioeconomic position is quite unique. Statistics Finland’s data on parents’ SEP were found for most of children whose height and weight data were available in the Avohilmo register. Our findings were in line with previous studies’ results on association between parents’ SEP and childhood obesity, confirming that linking data of administrative registers offers a possibility to monitor the prevalence of overweight and obesity in children according to the parents’ SEP. The use of registry-based data is cost-effective since health examination studies or questionnaires are not needed. This enables the development of the Finnish monitoring system on overweight and obesity in children [2] to also cover parents’ SEP, as well as the degree of urbanization of the municipality of domicile. To make this possible, further development is needed to make the linking of data from different administrative registers as flexible and up to date as possible.

### Strengths and Limitations

The main strength of the study is the large and comprehensive data from two administrative registers: the register of Primary Health Care Visits (Avohilmo) and Statistics Finland providing objective measures for both children’s height and weight and parents’ SEP. Parents’ SEP was found for almost all children (98%). Altogether, our data included measured height and weight and parents’ SEP for almost 200,000 children. As far as we know, there is no similar, large registry-based data available in other countries on both childhood obesity and parents’ SEP. Statistics Finland’s data on SEP is collected from various national registers, for example from the Population Register and the Income Register, making the data more comprehensive and reliable than data collected through questionnaires. The advantage of using register data is that there is no response-bias such as lower participation to surveys by people with obesity or low SEP [53]. In addition, there are not so many missing values, as there may be when asking children, or asking adults about information that they may consider sensitive, such as education and income.

However, there are also some limitations in the study. First, although the Avohilmo data collection has covered all outpatient primary health care delivered in Finland since 2011, the coverage of data on children’s height and weight was approximately 40% in 2018. Because most children and families attend to health check-ups and children are measured regularly, the low coverage of height and weight data is mainly due to problems with the electronic health records and technical data transmission in use [52]. The future challenge is to improve the coverage of Avohilmo data on the height and weight to it’s full potential, over 90%. It requires good collaboration between public health service providers, producers of patient’s electronic health records, and THL.

In addition, according to Statistics Finland’s data on adults living at the same address as a child, it is not always possible to verify if adults are parents, biological parents, or stepparents of the child. It is also difficult to ensure, whether the family is a nuclear family or a stepfamily. One limitation is also that Statistics Finland’s indicator “educational level of the highest qualification/degree” includes only degrees in secondary education or higher. However, very few people in Finland have no basic education. Therefore, if a person did not have a degree in secondary education, we assumed that the person had primary education. Another limitation is that the registry data used in the present study does not include information on health behavior such as diet and physical activity.

While no causal conclusions can be drawn from this cross-sectional study, our findings have important public health implications in Finland. The study showed that it is possible to develop the Finnish monitoring system on overweight and obesity in children to cover several aspects of parents’ SEP, as well as the degree of urbanization of the municipality of domicile. Our results also emphasize that primary prevention of obesity is essential, including identification of children at increased risk of obesity. Obesity prevention requires wide collaboration and a health-in-all-policies approach to improve children’s health and health equity.

### Conclusion

Parents’ low SEP, measured with several indicators, was associated with overweight and obesity in children in Finland.

Linking data of administrative registers on children’s growth and parents’ SEP is a potential, feasible and valid approach to monitor the prevalence of overweight and obesity in children by parents’ SEP.

## Abbreviations

Avohilmo, Register of Primary Health Care Visits; BMI, body mass index; SEP, socioeconomic position; THL, Finnish Institute for Health and Welfare.
