# Supplementary material for: Overweight and Obesity in Finnish Children by Parents’ Socioeconomic Position—A Registry-Based Study
Source: Int J Public Health. 2023 Sep 1;68:1605901. doi: 10.3389/ijph.2023.1605901 (PMC10502218; doi:10.3389/ijph.2023.1605901)
Supplement: Supplementary file 1 [file Table1.pdf]

Table S1. The prevalence of overweight in children, by age group, sex and socioeconomic position of parents, %. (A registry-based study, Finland, 2016–2018).

	Boys				Girls			
Age group (years)	2-6.99	7-12.99	13-15.99	16-17.99	2-6.99	7-12.99	13-15.99	16-17.99
N	34,814	36,229	17,270	11,903	33,459	34,619	16,720	9,409
Overweight[1,2]	14.2	35.4	30.8	29.0	13.3	28.7	27.6	25.4
Father's age (years)								
< 30	10.9	33.8	23.3	NA	11.5	32.1	26.5	NA
30-34	12.2	31.1	31.6	20.5	11.9	29.4	30.9	21.9
35-39	14.0	33.1	32.2	32.4	12.5	26.8	30.6	25.6
40-44	14.6	32.5	28.4	30.7	13.4	26.3	26.9	27.1
45-49	18.5	36.7	30.2	27.3	16.2	28.9	25.1	24.8
≥ 50	20.0	39.6	30.6	28.3	19.5	30.3	27.7	24.3
Mother's age (years)								
< 30	12.9	35.2	36.8	NA	12.8	32.6	43.3	NA
30-34	13.1	34.4	31.9	26.7	12.5	30.1	30.6	22.9
35-39	14.8	33.3	32.1	32.8	12.7	27.3	29.6	28.2
40-44	16.6	34.5	29.8	29.6	15.8	27.4	27.4	26.5
45-49	20.6	38.7	30.7	28.2	18.9	30.4	26.8	24.5
≥ 50	25.8	42.9	31.2	28.6	27.1	30.7	27.1	25.2
Household's disposable monetary income								

Low	15.1	36.3	31.7	30.6	13.6	30.6	31.1	28.1
Middle	14.0	35.4	31.0	29.3	13.2	28.5	27.1	25.3
High	11.2	27.3	24.5	22.8	10.8	21.3	20.2	19.8
Father's degree of highest education								
Low	15.9	41.8	36.9	33.3	14.5	34.2	34.6	29.3
Middle	15.4	36.9	34.1	32.1	14.6	31.5	29.7	28.6
High	11.1	29.2	23.5	23.1	10.2	22.3	21.7	19.5
Mother's degree of highest education								
Low	16.8	41.1	37.7	32.9	15.1	34.5	34.7	29.2
Middle	16.4	39.6	34.9	32.4	15.5	33.1	30.8	29.4
High	12.2	31.3	26.8	26.0	11.3	24.7	24.2	22.0
Municipality group								
Urban	13.1	34.0	29.1	28.1	12.1	26.9	26.8	24.6
Semi-urban	17.0	38.6	33.5	30.8	16.0	33.0	27.7	26.0
Rural	19.1	40.2	38.1	31.7	17.8	35.0	32.3	29.7

[1]overweight and obesity were defined according to the WHO growth reference for children (18,19)

[2] the prevalence of overweight includes obesity

Table S2. The prevalence of obesity in children, by child's age, sex and socio-economic position of parents, %.(A registry-based study, Finland, 2016–2018).

	Boys				Girls			
Age group (years)	2–6.99	7–12.99	13–15.99	16–17.99	2–6.99	7–12.99	13–15.99	16–17.99
N	3,4814	36,229	17,270	11,903	33,459	34,619	16,720	9,409
Obesity[1]	4.2	14.8	12.6	11.6	3.4	8.9	8.2	7.7
Father’s age (years)								
< 30	3.0	12.3	9.6	NA	3.5	10.6	8.8	NA
30-34	3.0	12.9	14.1	8.2	2.6	8.7	8.6	8.2
35-39	4.1	12.9	14.4	13.8	3.0	7.7	9.8	9.6
40-44	4.5	12.8	10.4	12.1	3.3	7.5	8.2	7.2
45-49	5.7	15.0	12.0	11.0	5.3	8.8	7.2	7.2
≥ 50	6.8	17.3	12.5	10.9	6.2	9.5	8.0	7.5
Mother’s age (years)								
< 30	3.8	16.7	21.1	NA	3.3	9.8	16.7	NA
30-34	3.8	14.0	14.2	12.2	3.0	9.5	10.6	7.3
35-39	4.2	13.5	13.6	12.3	3.1	8.0	10.2	10.4
40-44	5.0	14.1	11.2	12.8	4.9	8.4	8.0	8.3
45-49	6.8	16.9	12.4	10.7	5.4	9.7	7.5	6.8
≥ 50	6.1	18.9	13.5	11.6	10.2	10.4	7.4	7.9
Household's disposable monetary income								
Low	4.8	16.7	14.0	14.5	4.0	10.7	10.3	9.8

Middle	4.0	14.4	12.6	11.6	3.2	8.5	7.9	7.7
High	2.0	8.9	7.1	5.0	2.0	4.4	4.1	4.1
Father's degree of highest education								
Low	5.8	19.5	16.8	15.1	5.0	12.3	11.0	10.4
Middle	4.7	15.8	14.6	14.1	4.1	9.9	9.5	8.9
High	2.5	10.0	7.7	6.8	1.7	5.3	5.2	5.0
Mother's degree of highest education								
Low	6.2	21.3	18.0	16.2	5.1	13.5	11.1	11.1
Middle	5.3	18.1	16.1	15.1	4.6	11.3	10.7	10.1
High	2.9	11.3	9.2	8.5	2.3	6.4	5.9	5.6
Municipality group								
Urban	3.7	13.7	11.1	10.6	2.9	7.9	7.6	7.2
Semi-urban	5.5	17.2	15.2	14.3	4.7	11.0	8.7	8.6
Rural	6.1	18.5	17.6	13.6	5.4	12.5	11.4	9.4

[1]overweight and obesity were defined according to the WHO growth reference for children (18,19)
